# Medical students’ attitudes towards and views of general practice careers in Singapore: a cross-sectional survey and qualitative analysis

**DOI:** 10.1186/s12909-022-03298-7

**Published:** 2022-04-11

**Authors:** Yang Fang, Michael Soljak, Shawn Lien Ler Tan, Helen E. Smith

**Affiliations:** 1 Lee Kong Chian School of Medicine, Nanyang Technological University Singapore, 11 Mandalay Road, Singapore, 308232 Singapore; 2 Faculty of Medicine, Imperial College London, South Kensington Campus, London, SW7 2AZ UK; 3 Ministry of Health Holdings, 1 Maritime Square, Singapore, 099253 Singapore

**Keywords:** General practice, General practitioners, Family medicine, Primary care, Medical students, Medical school, Singapore, Career choice, Surveys and questionnaires

## Abstract

**Background:**

Like many other countries, Singapore needs to support its ageing population by attracting more doctors into general practice (GP) and family medicine (FM). To achieve this requires a better understanding of what attracts or deters medical students. We conducted a cross-sectional survey among medical students in Singapore.

**Methods:**

An online survey was distributed to students from all three medical schools to understand their likelihood of choosing primary care careers, what they valued in their careers, their attitude towards different aspects of general practice and family medicine relative to other medical fields, and the positive and negative perceptions of primary care held by themselves, their lecturers, and clinical mentors. They were able to elaborate the negativity encountered in the open-ended questions. Quantitative data was analyzed with descriptive statistics, principal component analysis, and linear regression; qualitative data was analyzed thematically.

**Results:**

The survey was completed by 391 students. Slightly over half indicated a likelihood of choosing a career in primary care. For their own careers, the students valued job satisfaction and career development opportunities the most. They perceived careers in primary care as being most likely to offer reasonable hours and close patient relationships, but least likely to offer career advancement potential relative to other medical fields. Their likelihood of choosing primary care careers was significantly predicted by what they value in their own career and their attitudes toward GP/FM relative to other medical fields, but not by the perceptions of GP/FM by others. Free-text responses illustrated how students encounter derogatory comments about GP/FM: the work being “mundane and repetitive”, the careers non-competitive, and the doctors poor in clinical competence.

**Conclusion:**

While the shortage of primary care doctors is a global issue, our findings highlight the value of situating inquiries in localized contexts. Medical curriculum should emphasize the critical role of primary care in the healthcare system and primary care doctors should be given due recognition to build a strong and motivated primary care workforce to serve the future healthcare needs of the population.

## Background

Like many other countries [1–3], Singapore needs to support its rapidly ageing population and the increasing need for chronic care in the community by boosting the number of doctors working in general practice (GP) and family medicine (FM). But to achieve this is not easy and medical schools have been urged to address these needs and students’ reservations to facilitate the recruitment of doctors into primary care [4]. The rich literature on this topic suggests medical students’ career preferences to be a complex product of individual characteristics, curriculum and training related factors, and larger environment for medical practice. Factors such as lower income [5], prestige [6], and even political climate [7], have been shown to dampen students’ interest in primary care careers. Medical schools play a substantial role in shaping students’ specialty preference in relation to GP/FM. Systematic and scoping reviews reveal that this can occur directly or indirectly through curricular design (e.g., lecture content, clinical rotations, exposure to patient groups) [8], presence of role models [9], and school environments that may be biased towards hospital care [10]. One study found that after completing a three-year preclinical curriculum, medical students have reported perceiving primary care physician to be more financially risky, less financially rewarding and prestigious, and to offer fewer career opportunities [11]. On an encouraging note, students reported being encouraged by early exposure to general practice in their course structure and the involvement of GP faculty in core teaching [12].

Besides medical education, specialty preference is also associated with factors intrinsic students’ background, interests, and values. Research has suggested a stronger preference for technical specialties in males [13], and stronger preference for specialty that offer more controllable lifestyles [14] and social orientation [13] (e.g., family medicine) in females. Having a parent who was a general practitioner was also associated with preferring a career in general practice [15]. In addition, having higher student debt was associated with less likelihood of choosing lower-paying specialties, such as primary care [16]. Still, academic interest and competency were identified as the most influential factors in specialty choice [17], and medical education is the key to developing these areas. While most of the literature consist of studies conducted outside of Asia, research within Asia have found higher income expectations [18] and poor awareness of general practice [19] to be associated with lesser likelihood to pursue this field of medicine.

To boost the number of doctors in the primary care workforce, Singapore has introduced over recent years many initiatives, including the opening of two new medical schools. In Singapore, general practice and family medicine is not recognized as a medical specialty and primary care doctors are regarded as generalists. To increase the number of doctors with a more generalist focus, the Ministry of Health has recently modified the fast-track North American style residency programs it introduced in 2010 as they have contributed to an overabundance of specialists. The proportion of residency openings in generalist fields has now been increased [20] and over 50% of residency positions offered in 2021 were in internal medicine or family medicine [21].

The career preference for specialist over generalist practice among medical students is a challenge for many countries [5, 15, 22, 23]. If increasing training opportunities for generalist practice in Singapore is to achieve its full potential, we need to better understand the factors that influence students’ career choice and their interest in primary care careers. Prior local studies of career choice of medical students have focused on factors such as finances [24] and sponsoring institute characteristics that affect their interest in specific specialties [25] and residency choices [26], with no attention to primary care. The surveys focusing on general practice come from overseas, from countries where the health ecosystems differ markedly from Singapore’s. Unlike many countries, family medicine is not recognized as a medical specialty in Singapore. Some earlier qualitative research we conducted with medical students highlighted several concerns about pursuing primary care careers (largely in comparison to specialist careers) including limitations in professional opportunities, dealing with mundane problems, lower perceived prestige, and hospital specialist mentors’ negative attitudes towards family doctors [27]. To better understand this data from focus group discussions and to quantify the frequency of the themes reported, we designed this cross-sectional survey of medical students in Singapore. Knowing what characteristics influence their career choice, and what might encourage or discourage their selection of primary care will help prioritize initiative to promote generalist practice.

## Methods

### Survey Design

The design of this survey questionnaire was informed by the existing literature [8, 17, 28], the findings of a series of focus groups with medical students in Singapore [27], and questions used in previous medical student surveys [7, 29]. The 19-item questionnaire was formatted with multiple choice and check-box selection questions, together with open-ended questions for free-text responses. Refinements were made after the questionnaire was piloted with six undergraduate medical students. The questionnaire can be found in the supporting documents.

The questionnaire focused on two areas: i) students’ career plans (what they value in their career, how likely they are to choose a career in primary care, their views on the opportunities and challenges in primary care, and the future of primary care in Singapore) and ii) the potential influence of other people on their career aspirations; how positively different groups of people view primary care (including university lecturers, clinical mentors, peers, family and friends, and the wider society), and whether they had encountered negativity to general practitioners and family physicians in the medical schools or hospital placements. If students had encountered negative comments about general practitioners, they were able to elaborate further in free text. In conclusion, the questionnaire asked about demographic characteristics (e.g., school, year of study, gender, age).

### Survey distribution

The survey was distributed on the survey platform Qualtrics to students in all years in the three medical schools in Singapore. We sought help from the offices of medical education and student life, teaching faculty, student societies, and personal links to distribute the survey link to the student body. The data collection took place between November 2019 and December 2020 having been delayed by the COVID-19 pandemic. Informed consent was obtained online, and no personally identifiable information was collected.

### Data analyses

Descriptive statistics were used to present an overview of the quantitative data. Where applicable, principal component analysis [30] was used to reduce questions with multiple items into a few dimensions to increase explanatory power. An overall multivariable regression analysis [31] was conducted to identify the factors that best predict medical students’ likelihood of pursuing primary care careers. All statistical analyses were conducted in SPSS Version 22 [32]. Free-text data underwent thematic content analysis [33].

Ethics approval was obtained from the Institutional Review Board, Nanyang Technological University (IRB-2018-07-007).

## Results

Five hundred and eighty-three students responded to the questionnaire (indicated by their completion of the first question on factors important to one’s own career) and two-thirds (*n* = 391) completed the survey in full. Based on the estimated medical student population of 2500, the response rate was 16%. The characteristics of the sample are shown in Table 1.

**Table 1:** Demographic characteristics of medical students

Demographic variable	N	Percent
School (M = 583)		
NUS Yong Loo Lin School of Medicine (NUS YLL)	241	41%
NTU Lee Kong Chian School of Medicine (NTU LKC)	265	45%
Duke-NUS Medical School (Duke-NUS)	77	13%
Clinical training phase (M = 583)		
Preclinical	296	50.8%
Clinical	287	49.2%
Age group (*N* = 391)		
Below 21	112	28.6%
21–25	233	59.6%
26–30	35	9.0%
31–35	10	2.6%
36–40	1	0.3%
Gender (*N* = 391)		
Male	186	47.6%
Female	195	49.9%
Prefer not to say	10	2.6%
Ethnic group (*N* = 391)		
Chinese	358	91.6%
Malay	2	0.5%
Indian	21	5.4%
Others	10	2.6%

There were approximately 1500, 640, and 320 students enrolled in NUS YLL, NTU LKC, and Duke-NUS, respectively, with an approximate response rate of 16, 41, and 24%. Across the schools, 41% of the students were in the pre-clinical and 59% of the students were in the clinical phases of their study

### Important factors in choosing one’s career

When considering the importance of different factors their career choice, the participants agreed most strongly that job satisfaction was important and least strongly that research opportunities was important. However, all items received a mean agreement score above the scale midpoint (3 out of a scale of 1 (strongly disagree) to 5 (strongly agree)), suggesting that overall, the items on their list were important to their career choice. The mean and SD of each item are shown in Table 2.

**Table 2:** Mean, SD of items and their loading on each component of “factors important to career choice”

	Mean	SD	Component loadings		
			1. Practical considerations	2. Style of working	3. career potential
Reasonable hours	4.25	0.80	.792		
Length of training	3.68	0.94	.703		
Location flexibility	3.62	0.97	.690		
Income potential	4.03	0.79	.434		
Community-based working	3.80	0.88		.811	
Teamwork	4.01	0.79		.718	
Close relationships with patients	3.98	0.82		.673	
Career development opportunities	4.39	0.66			.805
Research opportunities	3.02	1.06			.607
Professional status	3.90	0.79			.593
Job satisfaction	4.77	0.45			.492
Variance explained			22.6%	26.3%	14.1%

For Mean and SD, 1 = strongly disagree, 5 = strongly agree

A principal component analysis to explore the factors underlying the list of items revealed a three-component solution which explained 53% of the variance (Table 2). The Kaiser-Meyer-Olkin (KMO) measure of sampling adequacy is 0.680 and the Bartlett test of sphericity is significant (*p* < 0.05), suggesting sampling adequacy. The “practical considerations” component refers to consideration of potential investment of time and resources and the corresponding returns expected from a career. The items include reasonable hours, length of training, location flexibility, and income potential. The “style of working” component refers to how the work is carried out, particularly in relation to patients, colleagues and the working environment. The items include community-based working, teamwork, and close relationships with patients. The “career potential” component refers to the possibility that a career offers a variety of fulfilment. The items include career development opportunities, research opportunities, professional status, job satisfaction.

### Likelihood of choosing primary care careers

The students then rated how likely they were to choose primary care careers on a scale of 0 to 10. Across all schools and years of training, the mean and median likelihood of choosing primary care careers were 5.59 and 6.00 respectively, where 5 was the midpoint; 53.6% of all participants expressed a score of 6 or above. A one-way ANOVA showed that mean difference across the three schools was non-significant (F = 1.19, *p* = .305). Amongst the 402 students who indicated a likelihood of 5 or higher, 39% said that they had first considered this field before entering medical school (Table 3).

**Table 3:** First consideration of choosing primary care careers

	N	Percent
Before entering medical school	155	38.6%
Medical school (preclinical years)	100	24.9%
Medical school (clinical years)	139	34.5%
Missing data	8	2.0%
Total	402	100%

### Perception of primary care careers relative to other medical careers

In terms of what primary care careers are more likely to offer compared to other medical careers, reasonable working hours and close relationship with patients were rated the most highly, both receiving a score of higher than 4.5 on a scale of 1 (strongly disagree) to 5 (strongly agree), whereas research opportunities received the lowest ratings. Mean and SD of the items are shown in Table 4.

**Table 4:** Mean, SD of items and their loading on each component of “what GP/FM is more likely to offer than other medical professions”

	Mean	SD	Component loadings		
			1. Ambivalent attributes	2. Potential limitations	3. Potential strengths
Teamwork	3.42	0.99	.838		
Ample professional and collegiate support	3.49	0.88	.650		
Research opportunities	2.72	0.91	.636		
Career development opportunities	3.04	0.90	.576		
Opportunities to innovate	2.98	0.95	.551		
job satisfaction	3.65	1.02	.524		
Higher income	2.96	0.90		.937	
Professional status	3.05	0.93		.604	
Reasonable working hours	4.65	0.60			.794
Close relationship with patients	4.61	0.64			.747
Variance explained			32.4%	14.1%	10.6%

For Mean and SD, 1 = strongly disagree, 5 = strongly agree

A principal component analysis was used to explore the factors underlying the list of items revealed a three-component solution. The KMO measure of sampling adequacy is 0.776 and the Bartlett test of sphericity is significant (*p* < 0.05), suggesting sampling adequacy. The overall variance explained by the three components is 57%. (Table 4). The three components perceived of GP/FM compared to other medical careers are “potential strengths” (reasonable working hours, close relationship with patients), “potential limitations” (higher income, professional status), and “ambivalent attributes” (teamwork, ample professional and collegiate support, research opportunities, career development opportunities, opportunities to innovate, job satisfaction).

### Other peoples’ views of GP/FM

To understand how medical students’ attitudes toward primary care careers may be shaped by the views of others, we asked students to rate their perceptions of how positively different groups of people viewed GP/FM on a scale of 0 (extremely negative) to 10 (extremely positive). The students perceived most positivity from GPs they encountered on placements and family and friends who are GPs, and least positivity from doctors working in specialties and family and friends without medical background (Table 5).

**Table 5:** Mean, SD of items and their loading on each component of “other peoples’ views of GP/FM”

	Mean	SD	Component loadings	
			1. within the medical community	2. outside the medical community
Peers at medical school	5.33	2.04	.847	
Overall culture of medical school (including school curriculum)	5.68	2.20	.836	
Lecturers and trainers at medical school	6.17	1.88	.810	
Doctors on placements in other specialties	5.05	1.91	.762	
Family members and/or friends who have a medical background (non-GPs)	5.37	1.94	.654	
GPs on placements	7.09	1.81	.487	
General public	5.73	1.99		.886
Entertainment, TV (e.g., sitcoms, documentaries)	5.77	1.99		.848
Newspapers, online news articles or TV news	6.19	1.84		.801
Family members and/or friends (without medical background)	5.19	2.45		.733
Family members and/or friends who are GPs	6.72	2.00	.308	.337
Variance explained			43.6%	14.3%

For Mean and SD, 0 = extremely negative, 10 = extremely positive

A principal component analysis of the data found a two-component solution, which explained 58% of the variance (Table 5). The KMO measure of sampling adequacy is 0.840 and the Bartlett test of sphericity is significant (*p* < 0.05), suggesting sampling adequacy. The two components are groups “within the medical community” (peers at medical school, overall culture of medical school, lecturers and trainers at medical school, doctors on placements in other specialties, family members and/or friends who have a medical background (non-GPs), GPs on placements) and “outside the medical community” (general public, entertainment and TV (e.g., sitcoms, documentaries), news, family members and/or friends (without medical background)) The item “family and friends who are GPs” could not be reliably loaded onto either components in this solution.

### View of primary care’s increasing importance

To understand how the students view the future of primary care in Singapore, we asked participants to rate five statements pertaining to primary care’s increasing importance on a scale of 0 (least likely) to 10 (most likely). All statements received a score above the midpoint of 5, with “more doctors working in primary care” receiving the highest rating.

A principal component analysis was conducted to explore the factors underlying the list of items. A single-component solution emerged, suggesting that all five statements measured the same underlying construct. The Kaiser-Meyer-Olkin (KMO) measure of sampling adequacy is 0.819 and the Bartlett test of sphericity is significant (*p* < 0.05), suggesting sampling adequacy. The overall variance explained by the components is 58% (Table 6).

**Table 6:** Future of primary care

	Mean	SD	Loading on single component
More doctors will be working in primary care	7.80	1.52	.669
More care will be delivered in community settings	7.78	1.61	.849
GPs’ and FPs’ roles will be expanded	7.32	1.87	.798
Patients will increasingly opt for primary care settings for chronic care	6.91	1.99	.783
Career prospects in GP/FM in Singapore are improving	6.55	1.95	.701
Variance explained			58.2%

For Mean and SD, 0 = least likely, 10 = most likely

Finally, we regressed the primary outcome, the likelihood of pursuing primary care careers, on the potential predictors in the survey. The predictors other than age and gender were all scores derived from the results from the factor analyses presented above. The significant predictors in the final models were the importance of practical career considerations, importance of style of working, importance of career development potential as well as potential strengths and ambiguous attributes associated with primary care careers (Table 7). The model was significant (*R*^2^ = .31, *p* < .001). All the factors predicted the likelihood of choosing primary care careers positively with one exception, the importance of career potential to one's own career, which predicted likelihood negatively. The other predictors were non-significant.

**Table 7:** Overall model predicting the likelihood of choosing primary care careers

Predictor	Multivariable linear regression					
	p	Raw estimate	Standardized estimate	95% Lower limit	95% Upper limit	VIF
Intercept	.648	−.65				
Age^a^	.538	.09	.03	−.20	.39	1.05
Gender^b^	.530	−.13	−.03	−.53	.28	1.05
Practical considerations (own career)	**< .001**	.91	.23	.56	1.27	1.14
Style of working (own career)	**<.001**	.69	.19	.34	1.04	1.24
Career potential (own career)	**< .001**	−1.48	−.31	−1.91	−1.04	1.11
Ambiguous attributes (primary care careers)	**< .001**	1.11	.31	.74	1.49	1.42
Potential limitations (primary care careers)	.828	.03	.01	−.27	.34	1.39
Potential strength (primary care careers)	**.043**	.42	.09	.01	.83	1.11
Perceptions of GP/FM by groups in the medical community	.197	.11	.07	−0.06	.29	1.60
Perceptions of GP/FM by groups outside the medical community	.461	−.05	−.04	−0.19	.09	1.35
View of the future of primary care	.905	.01	.01	−0.17	.19	1.30

^a^Age was entered as a continuous variable

^b^There were nine participants who selected “prefer not to say” for gender. They were excluded in the analysis shown above. However, when included, the significance of all variables remained unchanged except for the gender variable. Those selecting “prefer not to say” had lower preference for primary care careers (M_prefer not to say_ = 2.44, compared to M_male_ = 5.71, M_female_ = 5.83, on a scale of 0 to 10). The reference level for gender was male

One third of students reported encountering negativity towards general practice and family medicine from medical school teaching staff, and 46% encountered it from hospital specialists. The percentage of students reporting the latter was significantly different across schools (*p* = 0.016), with 55% of NUS-YLL students, 38% of NTU-LKC students, and 43% of Duke-NUS students reporting having encountered negativity from hospital specialists. The negative comments were more frequently experienced in the clinical years of training compared to the preclinical years (*p* < 0.001). Derogatory comments were made about the nature of the work (“mundane and repetitive nature”, “boring”), the case mix (“dealing only with coughs and colds”, “Consultants say that GPs only treat easy acutes and manage chronics and it is not intellectually challenging”). It was portrayed as a non-competitive career option (e.g., “some mention how it’s the place doctors go to when they don’t want to continue IM residency and want something slack instead”, a “backup plan” or “last resort”). Anti-GP rhetoric extended to comments on poor knowledge (e.g., “useless, don’t know anything”, “GPs don’t know enough”), and poor performance in all aspects of clinical practice (e.g., “missing certain signs”, “incorrect administration of drugs or treatments”, “unnecessary referrals”).

## Discussion

Overall, the surveyed medical students demonstrated a fair amount of affinity toward primary care careers, with more than half indicating a score above the midpoint in their likelihood of choosing primary care careers. Amongst a list of factors commonly thought to be important to medical students’ career choice, job satisfaction was considered the most important [7, 34, 35]. Principal component analysis found three dimensions underlying the list of eleven items: practical considerations, style of working, and career potential. These results differed from that reported in a prior study conducted in Germany, where a principal component analysis revealed seven dimensions underlying a different list of 27 items [36]. Compared with other medical careers, the perceived strengths of primary care careers lie in reasonable working hours and close patient relationships and the perceived limitations were in higher income and professional status. How the remaining attributes listed compared to other medical careers were perceived to be ambivalent.

In terms of how different groups of people viewed GP/FM, the students reported that it was GPs on their placements who viewed GP/FM the most positively, while doctors they encountered from other specialties viewed GP/FM the most negatively. Other groups, such as the general public and teaching staff within the medical schools, scored somewhere in between. Principal component analysis found the different groups loading onto two factors: groups within the medical community and groups outside of the medical community.

### Comparison with existing literature

In comparison with other studies, the Singapore medical students’ attitudes toward primary care careers were relatively positive. Over half of the students indicated a likelihood above the scale midpoint of choosing primary care careers. A survey of students at Oxford University in 2017 found that 40% rated general practice careers as attractive or very attractive [7]. Surveys conducted elsewhere were less encouraging, but they focused only on top career choices; only 2% of medical students and recently qualified doctors in Brazil selected family medicine as one of their top three choices of medical career [15] and only 14% of Australian medical students indicated general practice as their top career choice [37]. In a survey of first year medical students across five Asian countries (China, Sri Lanka, Nepal, India and Malaysia) only 12.1% indicated an interest in pursuing general practice [38]. Compared to the survey by Barber and colleagues in 2018 [7], the students we surveyed attached more importance to career development opportunities and less to location flexibility. This difference may possibly be due to the small geographic size of Singapore (less than 750km^2^) which presents few accessibility problems. In other respects, attributes thought to be associated with primary care careers in comparison to other medical careers were fairly consistent across both surveys, as was the likelihood of choosing primary care careers. Similar to the Barber et al.’s finding that GPs were perceived to have lower status than hospital specialties, our findings also showed that GP careers were perceived to have lower professional status [39].

The regression results suggest that the students’ likelihood of pursuing primary care careers is primarily driven by their personal attitudes, such as factors they will value in their own professional life and their own perception of careers in primary care. The students appear less affected by external influences such as attitudes toward primary care held by teachers and mentors, friends and family, and the cultural milieu.

While some existing literature suggests that female students perceive family medicine careers more favorably [13, 40], in this study the two genders did not differ on their likelihood of pursuing GP/FM careers. Supplementary analyses suggest that compared to males, females perceived GP/FM careers to be more likely to offer professional status, research opportunities, and teamwork relative to other medical specialties; this difference is statistically significant (*p* < 0.05) but small in magnitude (mean difference of approximately 0.2 on a scale of 1 to 5). Likewise, older age did not correlate with higher likelihood of pursuing a GP/FM career, contrary to findings elsewhere [6]. This might be due to the limited age representation in our sample (88% of the sample were aged 25 or younger), which reflects the reality that the vast majority of medical students in Singapore begin their medical training at age 19.

### Strengths, limitations, and future directions

The strengths of this study include its generalizability for Singapore as it sampled students from both preclinical and clinical students’ years of study in all three medical schools, and thus presenting an overall picture of the potential local medical workforce. This is the first study using mixed methods and complements our earlier research that used only focus groups [27]. The study questions were largely drawn from validated items previously used in other surveys. This is one of the few studies that has employed factor analysis as a tool to understand what medical students’ value for their future careers and their perception of primary care careers. This statistical approach introduces more parsimony by identifying the conceptual dimensions underlying multiple items. The factors important in different countries may reflect the differences in country-specific characteristics and priorities.

Study limitations include relatively low response rates; despite many efforts to improve this, it was much lower than that reported for some other studies [41]. However, low response rates are not unusual for online surveys of this nature, and the COVID-19 pandemic also intervened. The response rates differed across medical schools and was understandably highest in the school in which the authors were based, which afforded more opportunities to promote the survey especially given the cross-institutional restrictions on physical contact during the pandemic. The completion rate among those who started the survey was also relatively low (67%). This may be due to its length (23% of those who completed the questionnaire took 15 min or longer) and some questions required considerable thought. We also observed the trend that those who were more likely to choose GP/FM were likely to have progressed further with the survey, suggesting disinterest in the topic to be a reason for exiting before completion. Given the cross-sectional design of our study, the findings are correlational and could be refined by longitudinal studies that track how medical students’ values and views change as they progress through their medical education. Understanding when the key decision points are for students’ career selection could inform the timing for primary care career promotion interventions.

### Policy implications

While the overall affinity towards primary care careers seemed positive in comparison to overseas reports, our findings identified several areas that may be counterproductive for attracting more new doctors into primary care, all of which can be addressed by policy. Several career attributes that appeared under the “ambivalent attributes” component (i.e., job satisfaction, career development opportunities, teamwork) received some of the highest importance ratings by medical students. To address this issue, the formal curriculum needs to be more explicit in articulating what can be expected from primary care careers with respect to these attributes. A recent scoping review suggests that early stage curriculum exposure, clinical placements, and role models are both positively related to interest in general practice [9]. Both the formal and informal curricula could be modified to enable students to gain more first-hand experience of these facets of general practice and family medicine, perhaps with greater opportunities for longitudinal patient follow up, more experience of managing multiple morbidity in a holistic manner, clinical attachments to other members of the primary care team, and greater involvement of general practitioners and family physicians in the teaching of clinical topics.

It is noteworthy that 39% of the respondents first expressed interest in general practice before they entered medical school, suggesting that nurturing students’ interest in general practice should begin in from the very early stages of their medical training. A systematic review of interventions aimed at increasing the proportion of medical students choosing primary care found that longitudinal programs were the only consistently effective intervention [42]. Positive role models can also play an important role; the evidence suggests that positive perceptions of the doctors the students work with during clinical placements are associated with more attractive ratings of a specialty and learning gained by students [43]. Positive role-modeling may occasionally backfire if student’s perceive the positive qualities (e.g., “genuinely smart”, “strong passion”) being displayed are not readily attainable by themselves [44]. Hence, role-modeling must be deliberately approached to generate interest, without suggesting the necessity of exceptional qualities.

Medical schools have a key role in preparing young doctors for work in the healthcare system of the future. Medical students who are academically rigorous but perceive general practice and family medicine as mundane need to realize that this is perhaps one of the most challenging branches of medicine with great potential and opportunities for innovation and research. To drive home the critical role of primary care in the healthcare system, the formal curriculum needs to better inform medical students of the patients’ journey between primary, secondary, and tertiary care, and the unique roles of each level of care. Teaching should be tailored to help students understand the breadth and complexity of general practice and family medicine, and the intellectual challenges of making early diagnoses and managing multimorbidity, using both physical and psychosocial approaches. Encouraging professional respect for GPs from all disciplines within medicine requires a top-down and bottom-up approach; from above, the medical colleges and councils need to promote professional behaviour, and from below, medical schools need to empower students to challenge denigration, encouraging students’ assertiveness skills to question and to formalize mechanisms for feeding back on serious disrespect encountered in the formal or informal curriculum [34, 35, 45].

Recognizing FM as a specialty may reduce the perception that GPs are not good enough to specialize. A recurrent challenge to GP/FM in Singapore has been the failure to recognize family medicine as a medical specialization, which persists even when Ministry of Health has recognized the urgent need to strengthen primary care as the foundation of its healthcare system. This combination of opposing concepts may inadvertently send the message to medical students that GP/FM is essential but unworthy of recognition, potentially deterring students with an interest to pursue this career pathway. In addition, portraying family physicians not just as clinicians but also having roles in healthcare policy, education and research would raise the profile of GP/FM.

## Conclusion

In 2017, the then medical services director of the Ministry of Health urged young doctors not to be in medicine for “prestige, financial rewards or fame”, not to choose “to be a super specialist when there is no such demand”, or to “choose a specialty primarily because it gives us a good work-life balance” [20]. Implicit in this message is the hope that young doctors will look beyond extrinsic rewards when choosing their career. Encouragingly, many students in this study have expressed interest in GP/FM that are independent of extrinsic considerations, and many have developed their interest in this field early in their undergraduate training. It is an important responsibility for undergraduate medical educators to promote and sustain this interest, with plentiful opportunities for students to discover and develop their interest in GP/FM. The recognition as specialists of those who undertake a residency program in Family Medicine and complete their Fellowship of the Singapore College of Family Medicine could contribute to addressing the apparent imbalance in status between primary and secondary care. Singaporean Ministry of Health’s strategy to achieve the “Three Beyonds” [46], beyond health care to health, beyond hospital to the community, and beyond quality to value, is heavily dependent on a strong, skilled and motivated GP/FM workforce for the future healthcare needs of the population.

## Data Availability

The datasets used in the study is available on reasonable request from h.e.smith@ntu.edu.sg.
